# Genome-wide scan for runs of homozygosity in South American Camelids

**DOI:** 10.1186/s12864-023-09547-3

**Published:** 2023-08-21

**Authors:** Stefano Pallotti, Matteo Picciolini, Marco Antonini, Carlo Renieri, Valerio Napolioni

**Affiliations:** 1 Genomic And Molecular Epidemiology (GAME) Lab, School of Biosciences and Veterinary Medicine, University of Camerino, Camerino, Italy; 2 SYNBIOTEC Laboratori s.r.l, Camerino, Italy; 3 Italian National Agency for New Technologies, Energy and Sustainable Development (ENEA), Roma, Italy; 4 School of Pharmacy and Health Products, University of Camerino, Camerino, Italy

**Keywords:** Alpaca, Llama, Vicugna, Guanaco, South American camelids, Runs of homozygosity, Signatures of selection, Domestication

## Abstract

**Background:**

Alpaca (*Vicugna pacos*), llama (*Lama glama*), vicugna (*Vicugna vicugna*) and guanaco (*Lama guanicoe*), are the camelid species distributed over the Andean high-altitude grasslands, the Altiplano, and the Patagonian arid steppes. Despite the wide interest on these animals, most of the loci under selection are still unknown. Using whole-genome sequencing (WGS) data we investigated the occurrence and the distribution of Runs Of Homozygosity (ROHs) across the South American Camelids (SACs) genome to identify the genetic relationship between the four species and the potential signatures of selection.

**Results:**

A total of 37 WGS samples covering the four species was included in the final analysis. The multi-dimensional scaling approach showed a clear separation between the four species; however, admixture analysis suggested a strong genetic introgression from vicugna and llama to alpaca. Conversely, very low genetic admixture of the guanaco with the other SACs was found. The four species did not show significant differences in the number, length of ROHs (100-500 kb) and genomic inbreeding values. Longer ROHs (> 500 kb) were found almost exclusively in alpaca. Seven overlapping ROHs were shared by alpacas, encompassing nine loci (*FGF5, LOC107034918, PRDM8, ANTXR2, LOC102534792, BSN, LOC116284892, DAG1* and *RIC8B*) while nine overlapping ROHs were found in llama with twenty-five loci annotated (*ERC2, FZD9, BAZ1B, BCL7B, LOC116284208, TBL2, MLXIPL, PHF20, TRNAD-AUC, LOC116284365, RBM39, ARFGEF2, DCAF5, EXD2, HSPB11, LRRC42, LDLRAD1, TMEM59, LOC107033213, TCEANC2, LOC102545169, LOC116278408, SMIM15, NDUFAF2* and *RCOR1*). Four overlapping ROHs, with three annotated loci (*DLG1, KAT6B* and *PDE4D*) and three overlapping ROHs, with seven annotated genes (*ATP6V1E1, BCL2L13, LOC116276952, BID, KAT6B, LOC116282667* and *LOC107034552*), were detected for vicugna and guanaco, respectively.

**Conclusions:**

The signatures of selection revealed genomic areas potentially selected for production traits as well as for natural adaptation to harsh environment. Alpaca and llama hint a selection driven by environment as well as by farming purpose while vicugna and guanaco showed selection signals for adaptation to harsh environment. Interesting, signatures of selection on *KAT6B* gene were identified for both vicugna and guanaco, suggesting a positive effect on wild populations fitness. Such information may be of interest to further ecological and animal production studies.

**Supplementary Information:**

The online version contains supplementary material available at 10.1186/s12864-023-09547-3.

## Background

Four species of South American Camelids (SACs) are distributed over the Andean high-altitude grasslands, the Altiplano, and the Patagonian arid steppes [1]. These include two wild species, vicugna (*Vicugna vicugna*) and guanaco (*Lama guanicoe*), and two domestic species, alpaca (*Vicugna pacos*) and llama (*Lama glama*) [1].

Vicuna (*Vicugna vicugna*) is a wild SAC producing a very fine fibre highly demanded by international market of luxury goods [2]. The wild population underwent a strong reduction due to intense hunting since the Spanish conquest: in the 1960s, the species was at risk of extinction as the number of specimens dropped from 2 million to less than 10,000 [3]. After decades of protection policies, vicugna population recovered [3], although controlled catches are still allowed.

The other wild SAC species is the guanaco *(Lama guanicoe*), a large herbivore whose population size declined from around 30 million to 5,000 specimens during the past century [4]. Such dropping started at the end of the 19th century when European sheep farmers colonized the Patagonia region killing both indigenous groups and guanacos due to their perceived role as competitors for sheep pasture [1]. Nowadays, guanacos concentrate in few low stock or sheep-free areas [1]. As occurred with the vicugna, preventive conservation strategies were adopted, although controlled capture and shearing of the wild animals are still allowed [4].

Alpaca (*Vicugna pacos*) is a SAC domesticated around 9,000 years ago and it has been associated with humans for as long as cattle, horses, and dogs [5]. Being an important resource for Andean highlands people, alpaca is breed by Peruvian farmer for meat and fibre production [6–8]. Several hypotheses were made concerning its ancestry [1]; indeed, it has been hypothesized that alpaca descended from the vicugna [9], the guanaco [10] or as hybrid between the vicugna and the llama [11].

The other SAC domesticated by indigenous communities is llama (*Lama glama*); this species was domesticated 5,000 years ago, starting from the wild guanacos [1]. Like the alpaca, these animals are breed for meat [12] and fibre production [13], being an important economic source for the Andean population.

Despite the extensive literature on SACs, most of the loci under selection are still unknown. Indeed, both natural and artificial selection shapes genetic variation across the genomes, rising the frequency of favorable alleles and specific haplotypes over time. This process leads to the establishment of genomic region with high differentiation across breeds and species known as “selection signatures”: the analysis of these regions allows to identify loci deviating from neutrality [14].

A well-established method to detect selection signatures is the genome-wide scan for Runs Of Homozygosity (ROHs): these are contiguous lengths of homozygous segments in the genome where the two haplotypes inherited from the parents are identical [15] and arise from a single common ancestor [16]. Genomic regions harboring selection signatures often overlap with shared ROHs regions within and across populations [17]. Moreover, the analysis of ROHs represents the state-of-the-art method for population inbreeding analyses [16].

Recently, important insights on SACs domestication using *F*_ST_ outliers and extended haplotype homozygosity analysis were provided by Fan and colleagues [11]. However, the identification of ROHs in SACs was never undertaken. This is mainly due to the lack of availability of genomic data. Indeed, recent efforts were only made to evaluate either the use of Bovine SNP-chip [18] or to design ad-hoc SNP-chip [19] for performing genomic studies in alpaca, thus further highlighting the need of genomic studies on SACs. However, the advent of next-generation sequencing led to an enormous amount of genomic data from several species, freely available for evolutionary and zootechnical research [20].

Starting from the whole-genome sequencing (WGS) data of 30 SACs retrieved from publicly available repositories, along with the *de novo* WGS of seven alpacas, we investigated the occurrence and the distribution of ROHs across the genome of SACs. Thus, we aim to unveil genomic selection signatures in the SACs, providing new information on the domestication and the potential convergent/divergent selection underwent by these species.

## Results

### Population structure

**Fig. 1:**
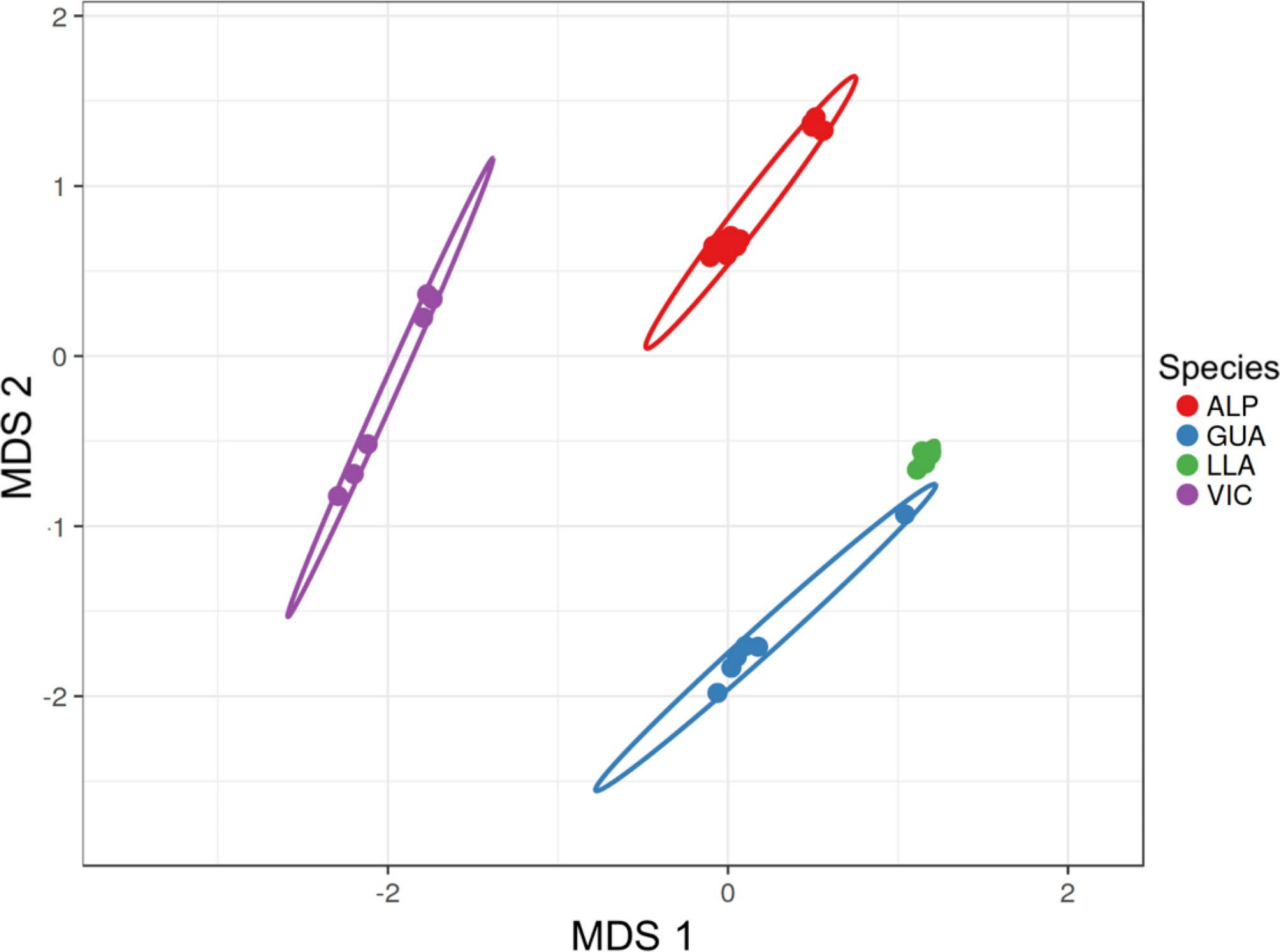
MDS plot of four South American camelids plotted for the first 2 dimensions. Each dot represents an individual sample (ALP, alpaca; GUA, guanaco; LLA, llama; VIC, vicugna)

Multi-dimentional scaling (MDS) was used to visualize the relationships between the 37 SACs included in the analysis; the plot showed a clear separation between the four species (Fig. 1).

**Fig. 2:**
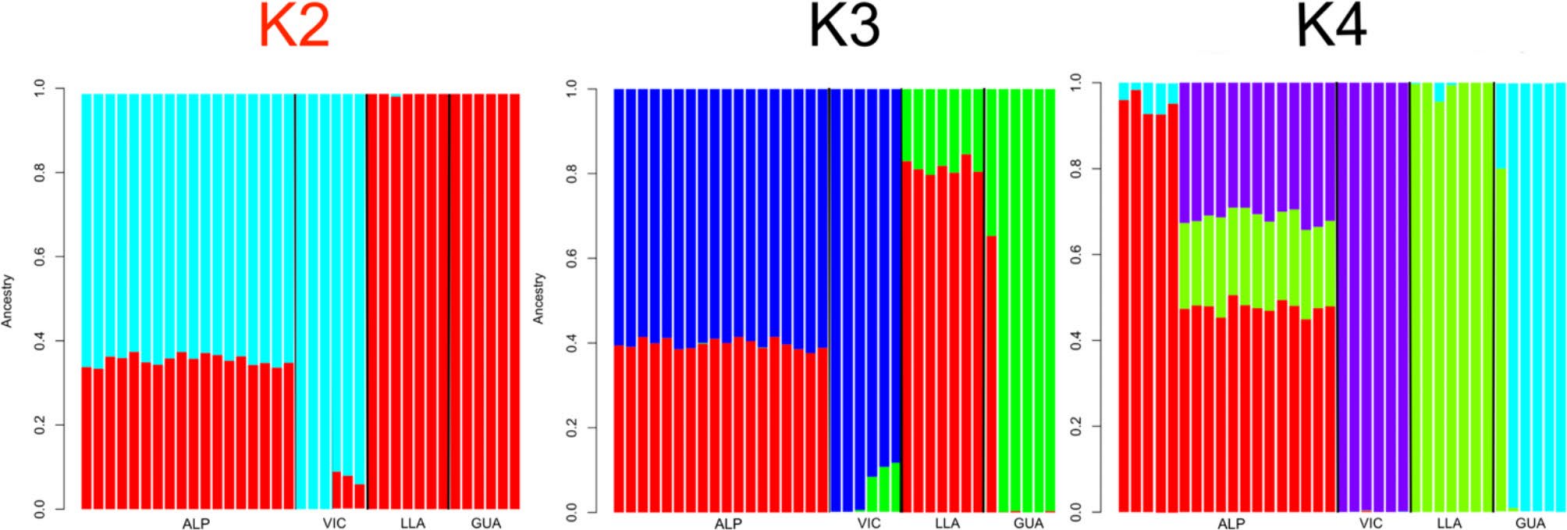
Admixture results for K = 2–4. The optimal number of clusters (K = 2), according to the cross-validation analysis, is indicated in red (ALP, alpaca; VIC, vicugna; LLA, llama; GUA, guanaco)

Admixture was computed by running the analysis for 2- to 4- clusters (K) (Fig. 2). The cross validation (CV) error test for each K value was performed to determine the most probable number of clusters. The CV errors were 0.648, 0.680, 0.751, and 0.846 for K = 2, 3, 4, and 5, respectively.

### Runs of homozygosity (ROHs) and genomic inbreeding (F_ROH_)

First, we evaluated the presence of ROHs with length 100-500 kb (Table 1). The average number of 100 kb ROHs found in the four species was 445 (± 175) for alpaca, 451 (± 287) for the guanaco, 315 (± 117) for llama and 489 (± 200) for vicugna. Concerning the length of the segments, alpacas showed the longest ROHs with an average length (in kilobases, kb) of 145,915 (± 15,960), followed by vicugna, (138,710 ± 2,630), llama (136,748 ± 2,840) and guanaco (134,830 ± 4,750). Thus, we evaluated the presence of longer ROHs (length > 500 kb) (Table 2); these were found only in 6 out of 18 alpacas, with an average length of 580,830 (± 53,62). Guanaco and llama did not show ROHs > 500 kb; however, only one vicugna showed a single ROH with a length of 517,702 kb. ANOVA did not show any significant differences in terms of ROHs number (P-value = 0.369) and length (P-value = 0.134) between the four species (Table 1).

**Table 1:** Distribution of ROHs and F_ROH_ (100-500 kb) in South American camelids

Species	N	Number of ROHs		Total kilobase distance spanned by segments			F_ROH_		Number of variants in the population	Segment size (kb)	
		mean	min-max	mean	min-max		mean	min-max		mean	min-max
*Alpaca*	18	445 (± 175)	264–803	67,477 (± 34,829)		31,605 − 141,769	0.043 (± 0.022)	0.020–0.090	25,526,240	145,915 (± 15,957)	133,186 − 176,550
*Llama*	7	315 (± 117)	218–566	61,733 (± 41,698)		28,622 − 78,747	0.028 (± 0.011)	0.018–0.050	14,153,719	136,749 (± 2,840)	131,292 − 139,651
*Vicugna*	6	489 (± 200)	267–772	43,226 (± 16,600)		36,585 − 107,703	0.043 (± 0.018)	0.023–0.069	17,145,639	138,705 (± 2,629)	137,024–143,715
*Guanaco*	6	451 (± 287)	208–986	68,092 (± 28,883)		26,548 − 140,133	0.039 (± 0.027)	0.017–0.089	14,793,242	134,826 (± 4,752)	127,633 − 142,122

N: number of animals

**Table 2:** Distribution of ROHs and F_ROH_ >500 kb in South American camelids

Species	N	Number of ROHs		Total kilobase distance spanned by segments		Segment size (kb)	
		mean	min-max	mean	min-max	mean	min-max
*Alpaca*	6	5,7 (± 4,89)	1–15	3,369.405 (± 2956,820)	508,712-8,987,700	580,829 (± 53,616)	508,712 − 651,219
*Vicugna*	1	1	-	517,702	-	517,702	-

N: number of animals

The inbreeding value based on ROHs > 100 kb was 0.043 (± 0.022) for alpaca and vicugna, 0.039 (± 0.027) for guanaco and 0.028 (± 0.011) for llama (Table 1). ANOVA did not show significant differences in terms of *F*_ROH_ values between the four populations (P-value = 0.399).

### Signatures of selection: overlapping ROHs

To identify potential signatures of selection, we focused on the ROHs shared by at least 70% of the population within each species (Table 3). Seven overlapping ROHs were shared by alpacas, with four located on chromosome NW_021964157.1 (chromosome 2), two on chromosome NW_021964193.1 (chromosome 17) and one on chromosome NW_021964178.1 (chromosome 12). These ROHs encompass nine loci (*FGF5, LOC107034918, PRDM8, ANTXR2, LOC102534792, BSN, LOC116284892, DAG1* and *RIC8B*). Nine overlapping ROHs were found in llama: three are located on chromosome NW_021964165.1 (chromosome 6), two on chromosome NW_021964196.1 (chromosome 19) and one in each of the remaining four chromosomes NW_021964160.1 (chromosome 3), NW_021964182.1 (chromosome 13), NW_021964193.1 (chromosome 17) and NW_021964195.1 (chromosome 18). A total of twenty-five loci were annotated for llama (*ERC2, FZD9, BAZ1B, BCL7B, LOC116284208, TBL2, MLXIPL, PHF20, TRNAD-AUC, LOC116284365, RBM39, ARFGEF2, DCAF5, EXD2, HSPB11, LRRC42, LDLRAD1, TMEM59, LOC107033213, TCEANC2, LOC102545169, LOC116278408, SMIM15, NDUFAF2* and *RCOR1*). 83% of the vicugna showed four overlapping ROHs; two of them were located on chromosome NW_021964156.1 (chromosome 1), one on chromosome NW_021964160.1 (chromosome 3) and on chromosome NW_021964175.1 (chromosome 11). Three loci were annotated (*DLG1, KAT6B* and *PDE4D*). Guanacos showed 3 overlapping ROHs located on chromosome NW_021964175.1 (chromosome 11), NW_021964178.1 (chromosome 12) and NW_021964223.1 (chromosome 34), respectively, with seven annotated loci (*ATP6V1E1, BCL2L13, LOC116276952, BID, KAT6B, LOC116282667* and *LOC107034552*).

**Table 3:** Signatures of selection: overlapping ROHs (≥ 70%) in each population

SPECIES	% of population that present the ROHs	N animals presenting the ROHs	Chr	Bp1	Bp2	Kb	nVariants	Genes
*Alpaca*	89	16	2 (NW_021964157.1)	47,004,014	47,097,954	93.941	1,864	*FGF5*, *LOC107034918*, *PRDM8*
	83	15	2 (NW_021964157.1)	47,223,830	47,288,964	65.135	1,095	*ANTXR2*, *LOC102534792*
	78	14	17 (NW_021964193.1)	32,857,799	32,908,888	51.09	743	*BSN*
	72	13	2 (NW_021964157.1)	47,109,781	47,116,834	7.054	119	---
	72	13	2 (NW_021964157.1)	47,301,062	47,372,080	71.019	1,462	*ANTXR2*, *LOC116284892*
	72	13	17 (NW_021964193.1)	32,961,329	32,990,407	29.079	297	*DAG1*
	72	13	12 (NW_021964178.1)	18,429,922	18,453,728	23.807	279	*RIC8B*
*Llama*	86	6	17 (NW_021964193.1)	28,016,567	28,120,792	104.226	1,256	*ERC2*
	86	6	18 (NW_021964195.1)	1,773,096	1,897,672	124.577	1,992	*FZD9*, *BAZ1B*, *BCL7B*, *LOC116284208*, *TBL2*, *MLXIPL*
	86	6	19 (NW_021964196.1)	20,312,362	20,426,143	113.782	1,814	*PHF20*, *TRNAD-AUC*, *LOC116284365*, *RBM39*
	71	5	6 (NW_021964165.1)	62,144,615	62,147,491	2.877	65	---
	71	5	19 (NW_021964196.1)	10,836,071	10,932,799	96.729	2,295	*ARFGEF2*
	71	5	6 (NW_021964165.1)	35,341,921	35,425,124	83.204	1,200	*DCAF5*, *EXD2*
	71	5	13 (NW_021964182.1)	8,027,836	8,128,031	100.196	2,000	*HSPB11*, *LRRC42*, *LDLRAD1*, *TMEM59*, *LOC107033213*, *TCEANC2*, *LOC102545169*
	71	5	3 (NW_021964160.1)	24,469,883	24,598,961	129.079	1,901	*LOC116278408*, *SMIM15*, *NDUFAF2*
	71	5	6 (NW_021964165.1)	62,072,906	62,144,562	71.657	1,065	*RCOR1*
*Vicugna*	83	5	1 (NW_021964156.1)	17,067,190	17,152,248	85.059	1,422	---
	83	5	1 (NW_021964156.1)	17,152,557	17,228,106	75.55	1,304	*DLG1*
	83	5	11 (NW_021964175.1)	7,005,163	7,020,809	15.647	231	*KAT6B*
	83	5	3 (NW_021964160.1)	25,397,227	25,469,962	72.736	1,116	*PDE4D*
*Guanaco*	83	5	34 (NW_021964223.1)	21,760,321	21,837,816	77.496	1,406	*ATP6V1E1*, *BCL2L13*, *LOC116276952*, *BID*
	83	5	11 (NW_021964175.1)	6,977,685	7,065,977	88.293	1,310	*KAT6B*
	83	5	12 (NW_021964178.1)	25,197,404	25,272,878	75.475	1,075	*LOC116282667*, *LOC107034552*

Chr: chromosome; Bp1: starting position of the segment; Bp2: ending position of the segment; Kb: segment length; nSNP: number of variants contained in the segment

The enrichment analysis failed to identify significantly involved molecular and functional pathways.

## Discussion

### Genetic relationships between the four South American Camelids species

Several studies have been performed to better understand the SACs ancestry and their genetic relationship, and diverse hypotheses on the potential crossbreeding between the four species have been made [1, 9–11].The MDS analysis showed a clear separation between the four species, with llama and guanaco close to each other respect to the alpaca and vicugna population. One guanaco clustered with the llama group. This was also observed in the admixture analysis suggesting a hybridization between the two species. To further study the population structure of SACs, admixture analysis was carried out. Recently, starting from WGS of eight alpacas, Fan and colleagues [11] found strong genetic admixture of the alpaca with vicugna and llama. In our study the ancestry of alpaca was studied considering 18 alpacas. By running admixture analysis for K2-4, the cross-validation error test suggested that K2 was the most probable value for K with alpacas clustering with vicugna and llama with guanaco. Moreover, the plot clearly showed a strong genetic introgression from vicugna and llama to alpaca. At K3 and K4 the plots revealed very low genetic admixture of the guanaco with the other species; guanacos in fact, showed a clearly separated gene pool. Only one guanaco showed genetic admixture with llama suggesting a possible hybridization between the two species as suggested by MDS plot. As already observed by Fan et al. [11], these results indicated alpaca as a possible hybrid species between llama and vicugna while not excluding the presence of a low genetic flow between guanacos and the other species.

### ROHs and genomic inbreeding

Applying different length criteria for ROHs detection can reveal information about population demography across a range of time frames, although the minimum segments length defined to identify a ROHs is yet set arbitrarily [21]. The similar amount of ROHs detected in the four species suggests a loss of genetic diversity of the wild species from a historical founder effect or genetic bottleneck [21] potentially due to the extensive hunting during the last century [1]; on the other, this data hint a low selective pressure for the two domestic species. Moreover, alpaca showed longer ROHs (100–500 kb), with an average length of 145,92 kb (± 15,96) compared to the other species where the average length ranged from 138,71 (± 2,63) to 134,83 (± 4,75). Although ANOVA did not show significant difference in the average length, probably attributable to the small sample size, the result suggests a more recent inbreeding for alpaca compared to other species [21]. This was further confirmed by the scanning for long ROHs (> 500 kb) which were detected exclusively in alpaca samples except for one vicugna which showed a single ROHs 517,702 kb long. Long ROHs are expected in populations that experienced recent inbreeding [22]. It should be stressed that even though the F_ROH_ values based on ROHs 100-500 kb were slightly higher for alpaca respect to the other species, ANOVA did not show any significant difference in terms of F_ROH_ values between the four population. The results suggest a low selective pressure in alpaca and llama whose inbreeding values are comparable to those of the wild populations. However further studies in a larger population are needed to validate the results.

### Overlapping ROHs: signature of selection

Genes under selection in SACs include loci selected for production traits (such as body conformation, fertility, and maternal traits) as well as for natural adaptation to the environment.

In alpaca, the overlapping ROHs showed loci potentially selected for adaptation to environment such as *BSN* and *RIC8B*, both involved in sensory processing of sound and olfactory signaling pathway [23–25] and stress related genes such as *ANTXR2*, *PRDM8* and *FGF5*. Signatures of selection on the latter three genes were already observed by Fan and colleagues [11], which suggested that *FGF5* and *ANTXR2* may play a key role in regulating hypoxia stress, while *PRDM8* was proposed as a novel gene associated with hypoxic adaptation. In addition, *ANTXR2* was also found under climate-mediated selection in human, sheep [26] and cattle [27, 28]. Not surprisingly, the signatures of selection in alpaca also include loci influencing reproduction efficiency and production traits such as fiber and meat quality. In this regard, *ANTXR2* was involved in reproductive regulatory processes in Bactrian camel [29], also influencing the development of primordial germ cells and reproductive organs, thus impacting on the age at first calving [30]. Similarly, *DAG1* affects fertility in dairy cattle being associated with sire conception rate [31] and regulation of spermatogenesis [32]. It must also be noted that signatures of selection included *FGF5*, a gene involved in the modulation of alpaca hair coat length [8]. Finally, alpaca genome was characterized by the presence of overlapping ROHs that harbor loci influencing meat attributes such as *RIC8B* [33, 34] and *DAG1* [28, 35].

In llama, most of the selection signatures encompasses loci involved in the regulation of reproduction and maternal traits: some are involved in the lipid metabolism during lactation, as *ARFGEF2* and *FZD9* which are known for their role in fatty acid transport [36] adipocytes differentiation [37] and quantity of milk produced [38]. Similarly, the *PHF20* gene is involved in milk production [39] and lactating efficiency [40], while *RCOR1* is a candidate gene implicated in lactose synthesis and milk yield [41]. Moreover, signatures of selections in llama suggested selection for efficient reproductive performances in harsh environments. *EXD2* for example, it has been involved in reproductive performance of cattle under heat stress [42]. In addition, overlapping ROHs comprise genes affecting pregnancy and fertility (*FZD9*) [43], heifer early calving (*PHF20* and *RBM39*) [44], resistance to uterine disease traits in first parity (*ERC2*) [45] and ovulation rate (*BCL7B*) [46]. Finally, signals of selection were found in *ARFGEF2*, a candidate gene associated with low fertility in single kid cashmere goat [47]. Like alpaca, signatures of selection for carcass quality traits were detected also in llama. In fact, *ARFGEF2* was found to be under selection in dromedary camel and proposed as determinant of camel body weight [48] while other selected loci such as *BCL7B*, *MLXIPL* and *NDUFAF2* are implied in fat deposition and depth [46, 49–52]. Two loci associated with animal domestication and behavior traits, were found under selection in llama. *ERC2* is involved in the neurotransmitter release, and it was shown to be related to behavioural changes such as reduction in fear [53]. Similarly, *BAZ1B* was recently associated with the domestication syndrome by influencing the development of the neural crest in Zebrafish [54]; moreover, the gene was also involved in the human self-domestication hypothesis as a master regulator of the modern human face [55]. Signal of selection for harsh climate condition in llama was found in genes involved in cellular response to heat stress (*HSPB11*) [56] and adaptation to UV exposure through pigmentation mechanism (*ERC2*) [57]. Finally, *LDLRAD1* is known to be potentially involved in resistance against bacterial infection [58], while *RBM39* has been proposed as target of putative selective sweep in swine being involved in RNA splicing and RNA processing [59].

As expected, the two wild species showed a lower number of loci under selective pressure (Table 3) and overlapping ROHs were identified only over three and four loci for vicugna and guanaco, respectively. Three genes under selection in wild SACs are known to be involved in reproduction. In the vicugna, *DLG1*, already proposed as putative signature of selection for reproductive traits in Iranian dromedary camels [60], was also associated with fertility in other species being implied in oocyte polarization during maturation in cattle [61] and ovary development and litter size in sheep [62]. Similarly, *ATP6V1E1* and *BCL2L13* were found to be under selection in guanaco; these genes are known to play an important role in mouse and cattle fertility [63, 64]. Four loci indicate adaptation driven by environmental conditions in wild SACs. In vicugna, overlapping ROHs in *DLG1* suggest selection for feeding efficiency in poor pasture quality [65] while the selection signals found in *PDE4D* hint the adaptation of the species to UV radiation exposure through the modulation of pigmentation and/or eye-protective phenotypes. Indeed, this gene was recently described as essential in signaling pathways of melanin encompassing signatures of selection for variation in pigmentation in Groningen White Headed cattle [66] and goat breeds from Southern China [67]. Potential environmental adaptation was also observed in guanaco as the species showed overlapping ROHs in *BCL2L13*, a gene selected for heat stress tolerance [68]. Environmental selection pressure for heat stress tolerance of the guanaco was also suggested by the presence of selection signals in *BID* gene that was found differentially expressed during heat stress in cattle [69–71] and buffalo [72]. Finally, it should be noted that both wild species present signatures of selection in *KAT6B*, a gene previously known to be associated with carcass traits and leg morphology in cattle [73]. A recent genetic association study suggested that a rare *KAT6B* haplotype is responsible for lower weight and height of the Russian Yaroslavl cattle, a breed adapted to the harsh conditions of the Yaroslavl region of eastern Russia. Authors suggested that the selected haplotype may be the result of an historical positive selection under the harsh environmental conditions and low feeding base and that the lack of such mutations in most other cattle breeds hints its negative effect in other environmental conditions and/or negative selection by humans [74]. Similarly, it can be assumed that environmental selective pressure has driven the shaping of guanaco and vicugna genome leading to the signature of selection detected in *KAT6B*. This assumption is also corroborated by the selective signal for high feeding efficiency [64] found in *DLG1* for vicugna.

### Limitations

The present study presents some limitations which must be discussed. First, the small sample size did not allow a wider identification of overlapping ROHs. We should acknowledge a paucity of publicly available genomic data on SACs, particularly for vicugna and guanacos, which are protected species, with their sampling being extremely difficult [3, 4]. Second, we considered exclusively the identification of a ROH island present in more than 70% of the species studied as signature of selection. However, further tests (e.g., Extended Haplotype Homozygosity, haplotype based FLK) are applicable to confirm the presence of real genetic selection. Further studies, on a larger sample size, will be necessary to confirm this preliminary evidence. Moreover, individuals from livestock population often showed high level of genetic relatedness which could led to population structure and bias in the estimation of the signature of selection. To reduce this bias, we filtered out from our sample individuals with identity-by-descent (IBD) PI-HAT ≥ 0.5. A recurrent limitation is the lack of consensus in establishing the criteria to define the ROHs [75] and the minimum segments length defined to identify a ROHs is often set arbitrarily [76]. In SACs these parameters were never defined. To overcome this concern, the parameters to detect the ROHs were set by averaging the results from previous work on livestock and pet species reviewed by Meyermans [16].

Finally, we should acknowledge that using sequence-based gene variants, coupled with a small sample size, may lead somewhat to false-positive genotypes since we could not apply a Hardy-Weinberg Equilibrium test that is a common procedure applied to filter out gene variants badly genotyped at genome-wide level. Nevertheless, our stringent quality control pipeline regarding the overall genotyping rate (> 99%) and the removal of related animals (IBD > 0.5), may somehow balance the pitfalls related to the sequence-based variant calling.

## Conclusions

Our results on SACs ancestry support the findings provided by Fan and colleagues [11] which demonstrated that alpaca genome showed signals of genetic introgression from vicugna and llama.

The identification of ROHs along the SACs genome showed a comparable level of genetic variability between the four species; however, population bottleneck can be assumed for vicugna and guanaco while low selection pressure, in terms of genetic inbreeding, was found for alpaca and llama.

The main aim of our work was to detect signatures of selection along the four SAC species. Not surprisingly, the identification of the overlapping ROHs showed divergent selection between the populations. Alpaca and llama hint a selection driven by environment as well as by farming purpose while llama and vicugna showed selection signal for adaptation to harsh environment. Interestingly, signatures of selection on *KAT6B* gene were identified for both vicugna and guanaco, suggesting a positive effect on wild populations fitness. Such information may be of interest to further ecological and animal production studies.

## Methods

### Sample collection

A total of fifty-five WGS were used for the study. Forty-eight samples were generated by previous projects (PRJNA233565, PRJNA340289, PRJNA512907, PRJNA612032 and PRJNA685331) and were retrieved from the NCBI Sequence Read Archive (SRA) (Supplementary Table 1). Seven in-house alpaca samples were sequenced *de novo*: skin biopsies were performed as described in Pallotti et al. [8]. Genomic DNA was isolated using the Genomic DNA Isolation Kit (Norgen Biotek Corp.), according to the manufacturer’s instructions. The library preparation was carried out at Genomix4Life (Salerno, Italy) using the Illumina DNA Prep Kit (Illumina) followed by a 150 bp sequencing at paired-end mode, using the Illumina NovaSeq 6000 System.

The final sample consisted of seven newly sequenced Peruvian alpacas and 48 publicly available WGS samples (28 alpacas, 7 llamas, 7 guanacos and 6 vicugnas). The public SRA files were downloaded to our server and converted to FASTQ file.

### WGS quality control

The quality of the FASTQ files was checked using FastQC [77] and the adapter trimming was performed with Trimmomatic [78]. Read pairs were mapped to the alpaca reference genome *VicPac3.1* [5] using Burrows-Wheeler Alignment MEM (BWA-MEM) [79]. The X chromosome and the unplaced-scaffold sequences were removed from the reference genome FASTA file before performing the alignment. Seventeen alpaca samples and one guanaco sample were removed because of a genotyping rate lower than 99%. The remaining 38 samples (19 alpacas, 7 llamas, 6 vicugna and 6 guanacos) were included in the study and used for further analysis. The sequence length ranged from 100 to 150 bp with a sequencing depth coverage rate ranging from 15 to 63X (Supplementary Table 1). BAM files were further processed using the Genome Analysis Toolkit (GATK, v3.4) [80] and the HaplotypeCaller method was used for variant calling. The resulting VCF containing the genomic variant calling of the 38 samples was converted to PLINK file using VCFtool [81].

To have independent samples and reduce the underlying population structure potentially biasing the estimation of ancestry and ROHs, subjects’ pairs with Identity-By-Descent (IBD) PI-HAT ≥ 0.5 were identified using PLINK 1.9 [82]; only one alpaca sample was removed due to PI-HAT value of 0.5. The remaining 37 samples (18 alpacas, 7 llamas, 6 vicugna and 6 guanacos) showed PI-HAT values ≤ of 0.125 and were used for the detection of ROHs.

### Population structure analysis

To perform population structure analysis, we removed from the joint called variant file all the variants with a minor allele frequency (MAF) < 5% and we pruned the remaining variants by Linkage Disequilibrium (LD) using the PLINK command “*--indep-pairwise 1,500 150 0.1*”. Starting from 40,360,774 variants, 20,557,058 variants were removed due to low MAF (< 5%) and further 19,769,677 variants were pruned due to high LD (r^2^ > 0.9). Finally, 34,039 variants and 37 samples were used to run the Multi-Dimensional Scaling (MDS) and the admixture analyses. MDS was performed using PLINK 1.9 [81]. The results were plotted using ClustVis [83]. Population structure analysis was performed on ADMIXTURE (Version 1.23) with K = 2, 3, and 4. The correct value for K was determined according to ADMIXTURE’s cross-validation procedure [84] and the results were plotted using R [85].

### Runs of homozygosity (ROHs) and genomic inbreeding (F_ROH_)

To detect ROHs, the dataset was not pruned for low MAF (< 0.5%) or high LD (r^2^ > 0.9), as suggested by Meyermans [16]. Since the criteria to detect the ROHs in SACs was never defined, we set the parameters by averaging the results from previous works on livestock and pet species reviewed by Meyermans [16]. Based on that, the following PLINK parameters were used: “*--homozyg --homozyg-kb 100 (or 500) --homozyg-snp 15 --homozyg-gap 500 --homozyg-window-missing 1 --homozyg-window-het 3”*. The “*--homozyg-window-het*” and the minimum length of 100 kb (“*--homozyg-kb 100*”) were chosen according to the information provided by Quinodoz [86] (2021) and Harder [87]. For the identification of overlapping ROHs we kept the ones overlapping in at least 70% of the sample, according to each species. All the loci in the selected ROHs were manually annotated using NCBI Genome Data Viewer (https://www.ncbi.nlm.nih.gov/genome/gdv/) starting from the genomic coordinates contained in the Plink files.

The inbreeding coefficient based on ROHs (F_ROH_) was computed as suggested by McQuillan [88] (2008) as:

$${F_{ROH}} = \frac{{{L_{ROH}}}}{{{L_{aut}}}}$$

where L_ROH_ is the total length of all ROHs in the individual’s genome, and L_aut_ is the length of the autosomal genome.

ANalysis Of Variance (ANOVA) was used in IBM SPSS Statistics 21 software to test for significant differences in the number and length of ROHs as well as for the F_ROH_ values between the four species, setting a statistical significance threshold of P < 0.05.

### Gene-based enrichment analysis

For each species, gene enrichment analyses were performed according to the genes encompassing the overlapping ROHs with the web-based tool Database for Annotation, Visualization, and Integrated Discovery (DAVID) v6.8 [89], which allows for the investigation of the Kyoto Encyclopedia of Genes and Genomes (KEGG) pathways [90] and Gene Ontology (GO) for biological processes [22].

### Electronic supplementary material

Below is the link to the electronic supplementary material.

Supplementary Material 1

## Data Availability

The VCF file from the *de novo* WGS of seven alpacas generated and analysed during the current study are available in the EVA (European Variation Archive) repository under the project PRJEB61878 [https://www.ebi.ac.uk/eva/?eva-study=PRJEB61878]. The forty-eight samples generated by previous projects and used in this study are available in the NCBI Sequence Read Archive (SRA) [PRJNA233565, PRJNA340289, PRJNA512907, PRJNA612032 and PRJNA685331].

## Abbreviations

WGS: Whole-genome sequencing
ROHs: Runs Of Homozygosity
SACs: South American Camelids
