# Supplementary material for: Genome-wide scan for runs of homozygosity in South American Camelids
Source: BMC Genomics. 2023 Aug 21;24:470. doi: 10.1186/s12864-023-09547-3 (PMC10440933; doi:10.1186/s12864-023-09547-3)
Supplement: Supplementary file 1 — Supplementary Material 1 [file 12864_2023_9547_MOESM1_ESM.docx]

**Supplementary materials.**

**Table S1. WGS sample description and exclusion criteria**

| **Batch** | **Species** | **SRA id** | **Sequence length** | **Coverage** | **Genotyping rate** | **N ROHs per sample** | **QC** |
| --- | --- | --- | --- | --- | --- | --- | --- |
| PRJNA233565 | Alpaca | SRR1552593 | 50 | 9 | 92.8 | - | Excluded because geno rate <99% |
| PRJNA233565 | Alpaca | SRR1552594 | 50 | 8 | 87.4 | - | Excluded because geno rate <99% |
| PRJNA233565 | Alpaca | SRR1552595 | 50 | 3 | 71.2 | - | Excluded because geno rate <99% |
| PRJNA233565 | Alpaca | SRR1552596 | 50 | 9 | 92.6 | - | Excluded because geno rate <99% |
| PRJNA233565 | Alpaca | SRR1552597 | 50 | 3 | 68.0 | - | Excluded because geno rate <99% |
| PRJNA233565 | Alpaca | SRR1552598 | 50 | 8 | 40.6 | - | Excluded because geno rate <99% |
| PRJNA233565 | Alpaca | SRR1552599 | 100 | 20 | 98.8 | - | Excluded because geno rate <99% |
| PRJNA233565 | Alpaca | SRR1552605 | 100 | 11 | 97.9 | - | Excluded because geno rate <99% |
| PRJNA233565 | Alpaca | SRR1552606 | 100 | 15 | 94.0 | - | Excluded because geno rate <99% |
| PRJNA233565 | Alpaca | SRR1552609 | 50 | 9 | 43.2 | - | Excluded because geno rate <99% |
| PRJNA233565 | Alpaca | SRR1552610 | 100 | 11 | 98.6 | - | Excluded because geno rate <99% |
| PRJNA512907 | Alpaca | SRR8616951 | 150 | 35 | 95.3 | - | Excluded because geno rate <99% |
| PRJNA612032 | Alpaca | SRR11905253 | 150 | 18 | 52.6 | - | Excluded because geno rate <99% |
| PRJNA612032 | Alpaca | SRR11905257 | 150 | 18 | 47.9 | - | Excluded because geno rate <99% |
| PRJNA612032 | Alpaca | SRR11905258 | 150 | 17 | 39.8 | - | Excluded because geno rate <99% |
| PRJNA612032 | Alpaca | SRR11905259 | 150 | 16 | 99.0 | - | Excluded because geno rate <99% |
| In house sample | Alpaca | - | 150 | 41 | 99.9 | - | Excluded because IBD ≥ 0.5 |
| In house sample | Alpaca | - | 150 | 62 | 100 | 680 | Included |
| In house sample | Alpaca | - | 150 | 42 | 99.9 | 798 | Included |
| In house sample | Alpaca | - | 150 | 50 | 99.9 | 612 | Included |
| In house sample | Alpaca | - | 150 | 37 | 99.9 | 235 | Included |
| In house sample | Alpaca | - | 150 | 51 | 99.8 | 638 | Included |
| In house sample | Alpaca | - | 150 | 43 | 99.8 | 747 | Included |
| PRJNA233565 | Alpaca | SRR1552607 | 100 | 15 | 99.4 | 251 | Included |
| PRJNA340289 | Alpaca | SRR4095110 | 100 | 23 | 99.7 | 319 | Included |
| PRJNA340289 | Alpaca | SRR4095135 | 100 | 23 | 99.7 | 263 | Included |
| PRJNA612032 | Alpaca | SRR11905254 | 150 | 18 | 99.7 | 475 | Included |
| PRJNA612032 | Alpaca | SRR11905255 | 150 | 19 | 99.7 | 373 | Included |
| PRJNA612032 | Alpaca | SRR11905256 | 150 | 18 | 99.7 | 366 | Included |
| PRJNA685331 | Alpaca | SRR13340600 | 150 | 63 | 99.8 | 361 | Included |
| PRJNA685331 | Alpaca | SRR13340601 | 150 | 63 | 99.9 | 371 | Included |
| PRJNA685331 | Alpaca | SRR13340602 | 150 | 62 | 99.8 | 397 | Included |
| PRJNA685331 | Alpaca | SRR13340603 | 150 | 59 | 99.9 | 386 | Included |
| PRJNA685331 | Alpaca | SRR13340604 | 150 | 62 | 99.8 | 383 | Included |
| PRJNA685331 | Alpaca | SRR13340605 | 150 | 59 | 99.8 | 312 | Included |
| PRJNA612032 | Guanaco | SRR11905252 | 150 | 15 | 93.9 | - | Excluded because geno rate <99% |
| PRJNA612032 | Guanaco | SRR11905249 | 150 | 20 | 99.5 | 984 | Included |
| PRJNA612032 | Guanaco | SRR11905250 | 150 | 19 | 99.5 | 400 | Included |
| PRJNA612032 | Guanaco | SRR11905251 | 150 | 19 | 99.5 | 206 | Included |
| PRJNA612032 | Guanaco | SRR11905261 | 150 | 18 | 99.6 | 249 | Included |
| PRJNA612032 | Guanaco | SRR11905272 | 150 | 18 | 99.5 | 528 | Included |
| PRJNA612032 | Guanaco | SRR11905273 | 150 | 19 | 99.5 | 321 | Included |
| PRJNA612032 | Llama | SRR11905246 | 150 | 17 | 99.5 | 220 | Included |
| PRJNA612032 | Llama | SRR11905247 | 150 | 16 | 99.5 | 565 | Included |
| PRJNA612032 | Llama | SRR11905248 | 150 | 22 | 99.6 | 306 | Included |
| PRJNA612032 | Llama | SRR11905268 | 150 | 18 | 99.6 | 235 | Included |
| PRJNA612032 | Llama | SRR11905269 | 150 | 19 | 99.6 | 278 | Included |
| PRJNA612032 | Llama | SRR11905270 | 150 | 20 | 99.6 | 269 | Included |
| PRJNA612032 | Llama | SRR11905271 | 150 | 18 | 99.6 | 323 | Included |
| PRJNA612032 | Vicugna | SRR11905260 | 150 | 21 | 99.7 | 678 | Included |
| PRJNA612032 | Vicugna | SRR11905262 | 150 | 20 | 99.7 | 262 | Included |
| PRJNA612032 | Vicugna | SRR11905264 | 150 | 18 | 99.6 | 313 | Included |
| PRJNA612032 | Vicugna | SRR11905265 | 150 | 16 | 99.6 | 445 | Included |
| PRJNA612032 | Vicugna | SRR11905266 | 150 | 16 | 99.6 | 451 | Included |
| PRJNA612032 | Vicugna | SRR11905267 | 150 | 18 | 99.6 | 767 | Included |
